# Validation of a Simple and Robust Liebermann–Burchard Colorimetric Method for the Assay of Cholesterol in Selected Milk Products in Ghana

**DOI:** 10.1155/2019/9045938

**Published:** 2019-10-13

**Authors:** Joseph K. Adu, Cedric D. K. Amengor, Naomi Kabiri, Emmanuel Orman, Stella Abla Gameli Patamia, Bernice Korkor Okrah

**Affiliations:** ^1^Department of Pharmaceutical Chemistry, Faculty of Pharmacy and Pharmaceutical Sciences, College of Health Sciences, Kwame Nkrumah University of Science and Technology (KNUST), Kumasi, Ghana; ^2^Department of Pharmaceutical Chemistry, School of Pharmacy, University of Health and Allied Sciences (UHAS), Ho, Ghana; ^3^Department of Chemistry, College of Science, Kwame Nkrumah University of Science and Technology (KNUST), Kumasi, Ghana

## Abstract

Cholesterol plays a key role in the synthesis of bile acids and steroid hormones in the human body. However, excessively high levels are usually implicated in cardiovascular diseases. For this reason, it is essential to monitor exposure to high levels of it in products meant for human consumption, and this calls for the need to develop analytical methods to detect them. The use of Liebermann–Burchard reaction in this study has been explored to develop a simple, reliable, and robust quantitative colorimetric method to assay cholesterol, and hence provide a good alternative to chromatographic methods. The developed method was validated and used to determine the contents of cholesterol in selected dairy products on the Kumasi Metropolis market. The method demonstrated a good linearity (*R*^2^ = 0.996) over concentration range of 0.01–0.08 mg/ml. It was also shown to be precise and robust. The limit of detection (LOD) and limit of quantification (LOQ) were determined to be 0.00430 mg/ml and 0.01304 mg/ml, respectively. Ten selected brands of canned milk (B1–B5) and fresh yoghurt products (A1–A5) were then assayed using the developed method. The results showed that three products from each category had cholesterol contents above the allowable content of 5 mg/100 g in dairy products. The study thus has proposed a simple colorimetric method that can be adopted by dairy products manufacturing facilities to rapidly determine cholesterol contents during manufacturing in order to monitor the safe consumption of their products, and eliminate or minimize possible future health hazards.

## 1. Introduction

Phytosterols constitute an important class of natural products that have been explored for their medicinal importance. Cholesterol, stigmasterol, and sitosterol are examples of phytosterols of which cholesterol is the most abundant in animals. Cholesterol is mostly found in animal fat such as milk, eggs, and cheese whilst stigmasterol and sitosterol are ubiquitous in plants. Cholesterol, however, is a starting material for the biosynthesis of bile acids, steroid hormones, and Vitamin D, which are also precursors for vital biological functions [1–3]. Dairy product consumption has been a daily routine for most people in both developed and developing nations usually as part of their diet. Milk consumption is associated with a reduced risk of noncommunicable diseases (NCDs) such as osteoporosis, colorectal cancer, and type 2 diabetes [4]. However, concern has been raised about possible association between high diary consumption and cardiovascular related diseases because of their cholesterol contents [5–7].

Amidst fears of cholesterol being a silent killer and a backbone for heart related diseases, there are high consumptions of milk and its products. The regulatory bodies together with the scientific community have, therefore, recommended the intake of lower fat dairy foods, to reduce the risks [8]. The intake of dairy products, thus, calls for close monitoring of their cholesterol levels to safeguard the health of the consumers. It is important then to screen and regulate the cholesterol content of dairy products on the market.

For some time now, a nonenzymatic HPLC method has been the routine and widely used analytical method for cholesterol detection and quantification in milk products [9]. Gas chromatographic methods as well have found use in its assay [1, 10, 11]. Preferably, using relatively simple, inexpensive, and readily available analytical methods will help achieve the same objective, especially in the developing countries; for the routine quality assessments of dairy products.

**Figure 1:**
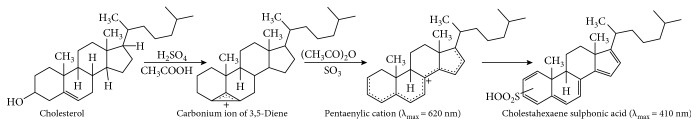
Reaction scheme for derivatization of cholesterol through Liebermann–Burchard reaction [15, 17].

Ultra–Violet Visible Spectroscopy is one of the most widely used techniques in pharmaceutical analysis [12]. Direct spectrophotometric method is used in the analysis of intensely absorbing analyte, while an indirect approach, which involves derivatization, is employed for weakly absorbing compounds. Cholesterol (cholest-5-en-3*β*-ol) has a weak absorbing chromophore, which can be derivatized through a Liebermann–Burchard reaction to obtain a strongly absorbing chromophoric moiety (Figure 1), referred hereinafter as Liebermann–Burchard product (LBP). This is considered simple, less-expensive, sensitive, and specific method, which prevents irrelevant absorption from other components in the matrix [1, 12]. The Liebermann–Burchard method, until this current study, had been employed for qualitative purposes [11], and so will be required to be optimized and validated for quantitative use; making it a reliable alternative to the well-established chromatographic methods [9–11, 13]. This study, therefore, aims to develop and validate a Liebermann–Burchard method for the assay of cholesterol and use it to assess the content of cholesterol in selected dairy products on the Ghanaian market.

## 2. Materials and Methods

### 2.1. Sample Collection

Commercial canned milk products and other yoghurt products (*N* = 10) were purchased from different retail stores in the Kumasi Metropolis, Ghana. Product selection was premised on the outcome of a survey conducted to establish which brands were consumed most and those products, which claimed low fat content or no fat present on their labels. Basic information on the products, including manufacturing and expiry dates, name of manufacturer, and origin were recorded. It was observed that most of the dairy products did not have Food and Drugs Authority's registration numbers on their labels (60% of yoghurts), which is a regulatory requirement. It was also noted that manufacturers only stated the content of fat in the product but not the cholesterol content. The samples were either analyzed immediately after being purchased or otherwise, stored at 2–8°C for future analysis.

### 2.2. Chemicals, Reagents and Glasswares

Fischer Scientific (United Kingdom) borosilicate glass volumetric flasks (10 ml, 100 ml and 250 ml, Grade A), pipettes (1 ml, Grade A), measuring cylinders (100 ml, Grade A), Erlenmeyer flasks with glass stoppers (25 ml, Grade B), separating funnel (250 ml, Grade B), beakers (500 ml, 100 ml, Grade B), glass funnel, petri dishes, and quartz cuvettes (square, 10 mm ± 0.01 mm path cell) were used for the study. All solvents used including acetic anhydride, concentrated sulphuric acid, chloroform, diethyl ether, methanol, and potassium hydroxide were of analytical grade and purchased from BDH Chemicals (BDH Limited Poole, England). Distilled water was produced In-house. Working standard of Cholesterol (C/5360/48, 95%) was purchased from Fissions Chemicals (United Kingdom).

### 2.3. Instrumentation

The colorimetric identification of cholesterol and subsequent analysis of dairy products was performed using a single beam Shimadzu UVmini-1240 UV Visible Spectrophotometer (Shimadzu Corporation, Kyoto, Japan), fitted with 10 × 10 mm cuvette holder, and scans within a wavelength range of 190–1100 nm produced from a Deuterium (D2) lamp and a Tungsten Halogen (WI) lamp. Data acquisition and results analysis were facilitated with Shimadzu UV Data Manager Software installed on a windows computer system. Analytical balance (Kern, Germany/WD140050809), melting point apparatus (Stuart Apparatus SMP10, UK), and a refrigerator (Whirlpool, Model WRT348FMEZ, USA) were among other equipment employed for the study.

### 2.4. Preparation of Stock Solution

A stock solution of the working standard, Cholesterol (1 mg/ml), was prepared by dissolving 0.1 g of it with chloroform in a beaker and transferred into a labelled 100 ml volumetric flask and made to volume. The solution was then kept refrigerated at 2–8°C for later use. Standard solutions for method development and validation were prepared by pipetting determined quantities of the stock solution and diluting to the required volumes with the same solvent.

### 2.5. Preparation of Liebermann–Burchard Reagent

The Liebermann–Burchard reagent (LBR) was prepared according to the method described in literature [14]. Briefly, 50 ml of acetic anhydride was pipetted into an amber glass vial and kept in an ice bath. After 30 minutes, 5 ml of concentrated sulphuric acid was pipetted and added carefully to the acetic anhydride in the vial.

### 2.6. Identification of Cholesterol

10 ml of 0.04 mg/ml cholesterol solution prepared from the stock solution was pipetted into a test tube and 2 ml of the LBR added and kept in the dark for 90 minutes. A control solution of chloroform and LBR was also prepared. Aliquots of the final solutions of cholesterol and blank were scanned within a wavelength range of 200–800 nm on the UV–Vis Spectrophotometer. The wavelength maxima (*λ*_max_) was recorded and compared with literature [15]. Results are shown in Table 1. The melting point of the purchased cholesterol was also determined and recorded (Table 1), in order to verify its authenticity [16].

### 2.7. Colorimetric Method Development

Cholesterol was dissolved in chloroform because it is a nonpolar compound (log *P* = 8.7) [18]. 2 ml of LBR was pipetted and added to a determined volume of the cholesterol solution and allowed to stand for 90 minutes for reaction to take place in the dark. After the reaction, the absorbance of the resultant solution was taken at 420 nm.

For the dairy products, a determined aliquot of the product was pipetted, saponified with 10% methanolic potassium hydroxide and separated in a solvent-solvent extraction with diethyl ether : water (5 : 2). The ethereal fraction, containing the cholesterol was then carried through the above-described procedure. The cholesterol content was estimated upon generating a linear calibration model from standard solutions of the working standard and inserting recorded absorbances of the samples into such model.

### 2.8. Analytical Method Validation

The developed method was validated in accordance with recommendations from the ICH guidelines [19], for specificity, precision, linearity and range, and robustness, and stability of the sample solutions.

#### 2.8.1. Specificity

**Figure 2:**
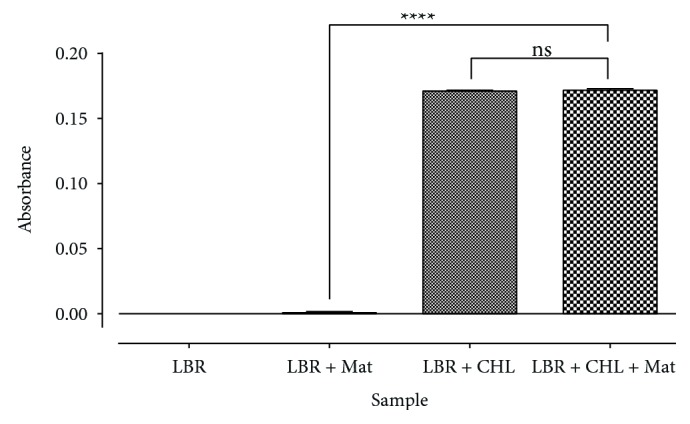
Test for specificity of method Data. analyzed by One-Way ANOVA followed by Tukey's post-hoc test at 95% confidence level.

The specificity of the colorimetric method was assessed by comparing the absorbances obtained from the chloroform solvent (placebo), the LBR only (blank), LBR with identified constituents of the matrix, LBR with cholesterol and then LBR with cholesterol and sample matrix. The results were then analyzed using ANOVA at 95% confidence level (Figure 2).

#### 2.8.2. Linearity, LOD, and LOQ

**Figure 3:**
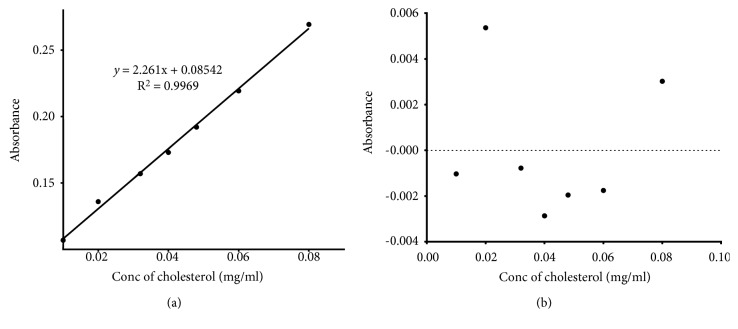
Prove of linearity of developed method.

In the test for linearity of the method responses, determined aliquots of the stock solution of the Working Standard (1 mg/ml) were pipetted to prepare standard solutions of concentrations ranging from 0.01–0.08 mg/ml using chloroform [19]. The absorbances of these solutions were recorded in replicates (*n* = 3), using LBR as the blank. Linearity was demonstrated from linear regression analysis (Table 2 and Figure 3(a)) and the residual plot of the absorbance against concentration (Figure 3(b)).

Detection limit (LOD), which is the smallest measured concentration of an analyte from which it is possible to deduce the presence of the analyte in the test sample with acceptable certainty [19] was determined from the slope (*S*) of the linearity plot and the standard deviation of the response at zero concentration level (*σ*) (Table 2). The slope was estimated from the analyte calibration curve (Equation (1)). The quantitation limit (LOQ), on the other hand, is the lowest amount of analyte in a sample that can be quantitatively determined with suitable accuracy and this was also determined from the slope and standard deviation of the response at zero concentration (Table 2).

#### 2.8.3. Precision

The precision of the method was demonstrated by determining both intra-assay (repeatability) and inter-assay (intermediate) precisions. The repeatability was proven by estimating the relative standard deviation (RSD) for ten replicate determinations of purity estimates at 100% concentration of the working standard (that is, 0.04 mg/ml) (Table 3). It was further demonstrated by determining the RSD of the purity estimates obtained over a concentration range (80%, 100%, and 120%) of the working standard (Table 3). The intermediate precision was proven by determining the RSD of results obtained by two analysts performing independent analysis on the same day and then, the same analyst performing tests on different days (Table 3).

#### 2.8.4. Accuracy

The accuracy of the method was established by comparing results obtained from purity estimate of cholesterol by the developed method, to the true value stated by the manufacturer [19]. The accuracy was further confirmed by calculating the percentage recovery of ten replicate values for various working concentrations (80%, 100%, and 120%) which is 0.032 mg/mL, 0.04 mg/mL, and 0.048 mg/mL, respectively (Table 4).

#### 2.8.5. Robustness

**Figure 4:**
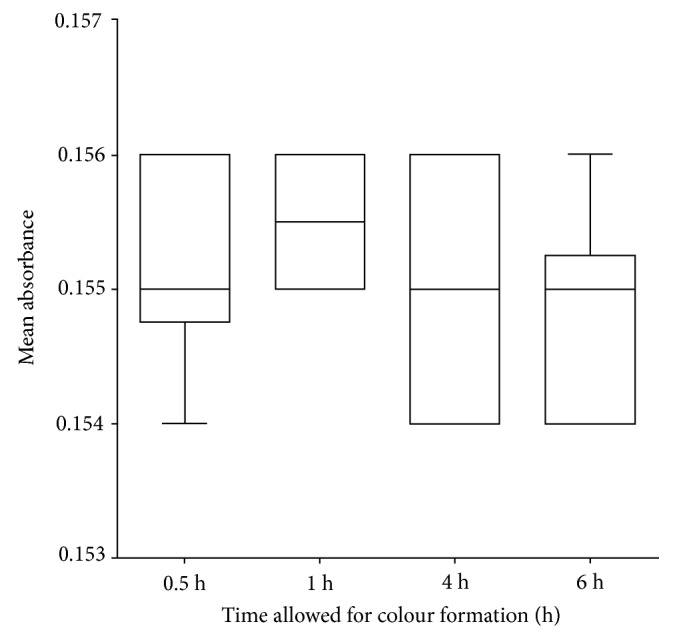
Box plot to illustrate robustness of developed method.

The robustness of the method was evaluated by investigating the change in time allowed for the Liebermann–Burchard reaction on the absorbance of the sample. The results were analyzed using One-way ANOVA (Table 5 and Figure 4).

#### 2.8.6. Stability of Solution

**Figure 5:**
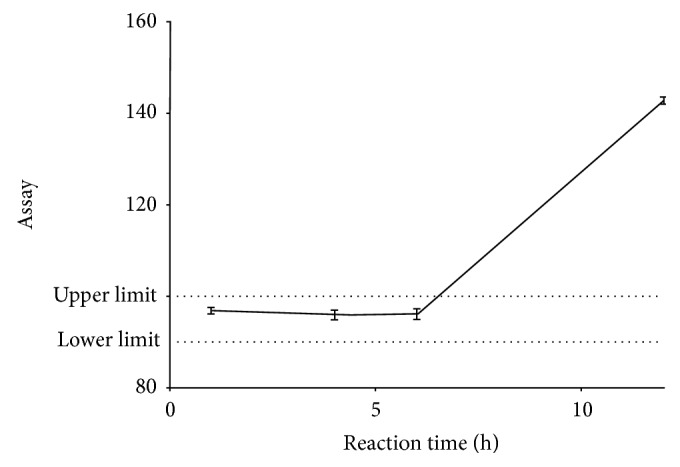
Stability profile of Fresh LBP prepared with 0.04 mg/ml of cholesterol as studied over 12 hours.

The stability of the LBP formation, which is critical for the analysis, was evaluated by preparing it with a 100% working concentration (0.04 mg/mL) of the working standard and taking replicate absorbances over 12 hours (Figure 5).

### 2.9. Extraction of Cholesterol from Test Samples

10 ml of each dairy product under investigation was independently pipetted and transferred into a stoppered 250 ml Erlenmeyer flask and saponified with 10 ml of 10% methanolic potassium hydroxide solution at 70°C for 30 minutes. The unsaponifiable fraction was then extracted with a mixture of diethyl ether and distilled water (5 : 2) in a separating funnel for three times. The ethereal fractions were transferred into petri dishes and left to evaporate to dryness to obtain the crystals of the compound.

### 2.10. Assay of Cholesterol from Dairy Products

The dried sample mass in the petri dish was redissolved in 20 ml of chloroform and transferred into a test tube. 2 ml of the LBR was added with shaking. The reaction was allowed to proceed for 90 minutes, with a dark green colouration indicating its completion. A blank solution of 20 ml of chloroform was kept for the determination. Triplicate absorbances of test and blank solutions were recorded and used to estimate the content of cholesterol. This procedure was repeated for all sampled dairy products.

### 2.11. Statistical Analysis

The results from the study were analyzed using GraphPad Prism 6 for Windows (Version 6.01, GraphPad Software, 2012). Test results were expressed as Mean ± SD and relative standard deviations (RSD), and also analyzed inferentially, using One-Way ANOVA (at 95% confidence level) to determine statistical differences in results generated. Results from the cholesterol assay in the sampled dairy products were also expressed Mean ± SD, and tested for statistical difference using Student *t*-test and One-Way ANOVA at 95% confidence level from SPSS Statistics (IBM Corporation, version 20, 2011).

## 3. Results and Discussion

### 3.1. Principle of the Colorimetric Assay

The colorimetric method developed for the assay of cholesterol in the dairy products was based on the ability of cholesterol, a weakly UV absorbing compound, to be derivatized into a strongly absorbing moiety at a wavelength (*λ*_max_) of approximately 420 nm [15] (Figure 1), void of sample matrix interferences in a reaction known as the Liebermann–Burchard Reaction [20]. Cholesterol, which was identified accordingly (Table 1), in the presence of concentrated sulphuric acid and acetic anhydride, was oxidized to a conjugated pentaene known as cholestapolyene carbonium ion [15, 21] and this undergoes further reaction to form cholestahexaene sulphonic acid, with wavelength of absorption (*λ*_max_) of 410 nm [15, 17] (Figure 1). LBR, containing acetic anhydride and concentrated sulphuric acid, provided the necessary conditions for the reaction to proceed. The quantitative conversion of all cholesterol to LBP provided the basis for the colorimetric estimation of its content. In the dairy products, however, saponification was carried out to convert all cholesteryl esters and glycosides to free cholesterol, which was then extracted into the ethereal fraction in the solvent-solvent extraction.

### 3.2. Validation of Developed Colorimetric Method

The developed method was showed to be specific towards cholesterol (Figure 2). In addition, it was observed that the absorbance of LBP from a reaction of cholesterol and LBR was significantly different from that of LBR, sample matrix and chloroform (*F*_(3, 16)_ = 72033; *p* < 0.0001; *q* = 483.1; *n* = 5). The sample matrix was shown not to contribute significantly to the absorbance (*q* = 1.697). The method has been demonstrated to have a suitable level of precision (Table 3), accuracy (Table 4), and linearity within a concentration range of 0.01–0.08 mg/ml, with an LOD of 0.00430 mg/ml and LOQ of 0.01304 mg/ml (Figure 3 and Table 2). It was also robust (Figure 4 and Table 5). The LBP was shown to be stable within 6 hours of preparation (Figure 5). This indicated that, the prepared solution is recommended to be used within 6 hours of preparation.

### 3.3. Analysis of Dairy Products

**Figure 6:**
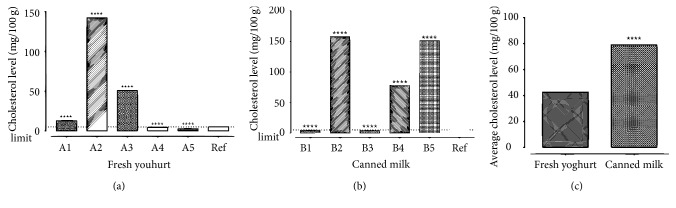
Cholesterol contents in sampled products.

The results from the experiment show that all the samples contained sterols, particularly, cholesterol, as they all reacted with LBR to produce derivatized products subsequently analyzed at 420 nm. The cholesterol levels in the fresh yoghurt products ranged between 2.620 ± 0.0511 mg/100 g (Brand A5) and 142.20 ± 0.000 mg/100 g (Brand A2), while the levels in the canned milk products were between 4.390 ± 0.0255 mg/100 g (Brand B1) and 157.9 ± 0.0675 mg/100 g (Brand B2). 60% of both yoghurt products and canned milk products were observed to have high cholesterol levels above the dietary daily requirement of cholesterol in milk products (5 mg/100 g) recommended by United States Department of Agriculture Product. A2 did not contain any information on the constituents, making it difficult to justify the high levels. In the case of the B2, it was noted that the product label claimed the presence of soya base, a plant-derived dairy source in the product. It could be argued that the high levels recorded may be due to the presence of cholesterols and other phytosterols like sitosterols and stigmasterol [22]. It was generally observed that cholesterol levels in the canned milk products were higher than that in the fresh yoghurts (*t* = 2426, *df* = 4;*p* < 0.0001; Figure 6(c)). This could be attributed to animal fats used; either as whole milk [23] or mainly as milk solids [24]. Whole milk is thought to contain 26%–42% of fats, and so possess high contents of cholesterol [23]. The use of such sources is expected to result in high cholesterol levels, as seen in B5 (151.3 ± 0.0442 mg/100 g). However, milk solids and milk solid nonfats have proven better alternatives, although the latter is the most preferred health wise. The use of milk solids, which contain fats [25] also would account for high cholesterol levels and so would require a defatting process during manufacturing of the dairy product. In the absence of that, it could result in high cholesterol levels in the final product, as was in the case of B4 (77.82 ± 0.000 mg/100 g). On the other hand, a thorough defatting process would lead to low cholesterol levels, as evident in B3 (3.829 ± 0.0442 mg/100 g). The use of milk solid nonfat is advocated by a number of researchers, especially for its low level of cholesterol. Its use in B1 could thus account for the low cholesterol level observed (that is, 4.390 ± 0.0255 mg/100 g). Generally, it was observed that either low milk fats or skimmed milk was used in the manufacture of the yoghurt products, thereby accounting for their relatively low cholesterol levels.

Insufficient information on the products such as lack of product registration numbers and also the fact that manufacturers only stated the fat content of the product and not the cholesterol content left consumers with no choice. Information on the cholesterol levels in such products if present, would guide different people with different health needs to make informed choices of dairy products to patronize.

## 4. Conclusions

It has been demonstrated that the Liebermann–Burchard reaction *via* colorimetric method could be used in successful analysis of cholesterol in dairy products. It serves as a reliable and robust alternative method to currently employed chromatographic methods which are expensive. From the sampled dairy products analyzed, it has been shown that canned milk products contained more cholesterol than yoghurt products and that most of the manufacturers adopted milk solids and milk solids nonfat as their dairy source for the manufacture.

## Figures and Tables

**Table 1:** Confirming the identity of cholesterol standard.

	Melting point	Wavelength maxima (*λ*_max_)
Working standard (cholesterol)	148°C–150°C	420 nm
Reference	147°C–150°C [16]	410 nm [15, 17]

**Table 2:** Table showing results from test for linearity test, LOD, and LOQ.

Parameter	Values
Slope	2.200 ± 0.02914
*Y*-intercept when *X* = 0.0	0.08542 ± 0.001368
*X*-intercept when *Y* = 0.0	−0.03778
1/slope (*S*^−1^ )	0.4422
*R* square	0.9969
Sy.x (*σ*)	0.002948
LOD	$\frac{3.3\sigma }{S}=0.00430\;\text{mg}/\text{ml}$
LOQ	$\frac{10\sigma }{S}=0.01304\;\text{mg}/\text{ml}$

**Table 3:** Results showing precision of results from the developed method.

Precision parameters			Mean absorbance ± SD	RSD
Intra-assay precision	*Outcome from 10 replicate determinations*		95.97 ± 1.016	1.06%
	*Triplicate determinations from three different concentrations*	80%–0.032 mg/ml	96.88 ± 0.7221	0.75%
		100%–0.040 mg/ml	95.97 ± 1.016	1.06%
		120%–0.048 mg/ml	96.83 ± 0.7773	0.80%

Inter-assay precision	*Same analyst*	Day 1	94.25 ± 0.9697	1.03%
		Day 2	96.05 ± 1.516	1.58%
		Day 3	93.01 ± 1.314	1.41%
	*Different analysts*	Analyst 1	94.53 ± 0.8770	0.93%
		Analyst 2	95.91 ± 1.695	1.77%
		Analyst 3	93.42 ± 1.306	1.40%

			*Acceptance criteria*	<2%

**Table 4:** Results showing accuracy of results from the developed method.

Test concentration (mg/ml)	Mean% recovery ± SD	Test concentration (mg/ml)	Purity estimate from analysis
0.032	102.0 ± 0.75889	0.032	96.88 ± 0.7221
0.040	100.9 ± 1.017	0.040	95.97 ± 1.016
0.048	102.1 ± 0.6654	0.048	96.65 ± 0.7527

*Acceptance Criteria*	[98%–102%]	*True assay value (by manufacturer)*	95%

**Table 5:** Robustness of developed method at different times for Liebermann–Burchard reaction.

	0.5 h	1 h	4 h	6 h
*Mean Absorbance ± SD*	0.1551 ± 0.0007379	0.1555 ± 0.0005270	0.1549 ± 0.0008756	0.1548 ± 0.0007888
*RSD*	0.48%	0.34%	0.57%	0.51%
*One-way ANOVA*	*F*_(3,36)_ = 1.734; *p* = 0.1775			

## Data Availability

All data are available in the Department of Pharmaceutical Chemistry, Faculty of Pharmacy and Pharmaceutical Sciences, College of Health, Kwame Nkrumah University of Science and Technology (KNUST), Kumasi, Ghana. as raw data stored in the instruments and also in the researchers report.
